# The fundamental role of storytelling and practical wisdom in facilitating the ethics education of junior doctors

**DOI:** 10.1177/1363459319889102

**Published:** 2019-11-18

**Authors:** Alexis Paton, Ben Kotzee

**Affiliations:** University of Leicester, UK; University of Birmingham, UK

**Keywords:** empirical ethics, medical ethics education, narrative, phronesis, practical wisdom, qualitative, sociological bioethics, storytelling, United Kingdom

## Abstract

Practical wisdom is a key concept in the field of virtue ethics, and it has played a significant role in the thinking of those who make use of virtue when theorising medical practice and ethics. In this article, we examine how storytelling and practical wisdom play integral roles in the medical ethics education of junior doctors. Using a qualitative approach, we conducted 46 interviews with a cohort of junior doctors to explore the role doctors feel phronesis has in their medical ethics practice and how they acquire practical wisdom through storytelling as an essential part of their medical ethics education. Through thematic analysis of the interviews, we discuss the key role storytelling about moral exemplars and role models plays in developing medical ethics education, and how telling stories about role models is considered to be one of the most useful ways to learn medical ethics. We finish by developing an argument for why practical wisdom should be an important part of medical ethics training, focusing on the important role that phronesis narratives should have in teaching medical ethics.

## Introduction

*Phronesis* – or practical wisdom – is a key concept in the field of virtue ethics (Russell, 2009), and it has played a significant role in the thinking of those who make use of virtue when theorising medical practice and ethics. In recent years, a large body of literature has not only recognised the importance of moral virtues like care, honesty and courage to medical practice but also argued that the ethical doctor embodies a practical moral know-how, something that is necessary if good moral motivations (dispositions or virtues) are to produce or result in good clinical practices. This practical moral know-how is often referred to or represented as ‘professionalism’, ‘professional judgement’ or ‘clinical judgement’. Drawing on Aristotle, a number of virtue ethicists concerned with medicine and healthcare (e.g. Kaldjian, 2014, 2010; Pellegrino and Thomasma, 1993) have called this know-how ‘phronesis’. While the importance of the virtue of *phronesis* is a well-established feature of the literature in medical ethics (Kotzee et al., 2016), little is known about the extent to which *phronesis* is in fact present in medical practice, and even less work has been done to understand how doctors *acquire* the virtue of phronesis.

In this article, we examine the role doctors feel phronesis has in their medical ethics practice. Drawing on Zagzebski’s (2013) recent ‘exemplarist’ account of the acquisition of *phronesis*, we focus on the role storytelling plays in developing phronesis. Using a hybrid methodology that draws on the social sciences and the humanities, we outline how the philosophical concept of phronesis can be examined empirically and sociologically. We highlight how phronesis is considered an important part of best practice, and how storytelling played an integral role in the development of phronesis during the medical training of a cohort of junior doctors. We argue that not only is storytelling one of the primary ways that phronesis is developed, but it is also the primary way that the development of phronesis can be examined empirically.

## What is phronesis and why is it important to ethical decision-making?

In the *Nicomachean Ethics*, Aristotle sets out an account of morality in terms of the moral virtues, focusing on the traits of a person’s moral character. The moral virtues are not innate features of a person’s personality but are acquired dispositions or habits (NE 1105b25–6, 1999). These are learned over time from moral role models (often one’s teachers or parents) and are reinforced through experience and moral practice. Thus, the ‘honest’ person is one who has become habituated to acting honestly, the ‘kind’ person is one who has acquired the habit of acting kindly, and so on. However, an account of morality sketched merely in terms of labelling moral virtues is incomplete, for two prominent reasons. First, being habitually ‘kind’ does not always make clear what a person should do in a particular situation, such as those where kindness may result in a negative outcome. For instance, a doctor may find themselves pulled in two directions by their kindness – is it more kind to prolong a terminally ill patient’s life or is it more kind to relieve pain at the cost of a few days’ life? Second, different virtues often conflict with one another. The doctor who is *both* kind and honest, for instance, may find that being honest in a particular situation comes at the cost of kindness (or vice versa) – take the decision whether to tell a patient the unvarnished truth about a tough prognosis, or sparing their feelings by sugar-coating the truth. For this reason, Aristotelian accounts of virtue ethics stress that, in order to *implement* virtuous action, the good doctor must not only have these moral virtues but also needs a further virtue – the intellectual virtue of *phronesis* – to know *how* to act virtuously in any given situation (Kristjánsson, 2007, 2015; Moss, 2011; Pearson, 2007; Russell, 2009).

A large literature has built around the role that *phronesis* plays in understanding how doctors make ethical decisions. Much of this literature presents *phronesis* as central to the skill of clinical judgement, as the ability of the good doctor to weigh up all the relevant factors that go into a diagnosis and treatment plan and to come to a well-balanced decision (Kotzee and Ignotwicz, 2015; Kotzee et al., 2016). Virtue ethicists like Pellegrino and Thomasma (1994), Schultz and Carnevale (1996), Montgomery Hunter (2006), Kaldjian (2010, 2014) and Toon (2014) also argue that the virtue of *phronesis* is crucial to good ethical decision-making in medicine. While virtues ensure that people act from the right intentions, taking the action itself requires practical moral know-how to determine how the right course of action can be implemented or pursued. This kind of practical wisdom is not easy to acquire. It develops over time and requires long practice and learning from example (Kassam, 2010). For many who study virtue ethics (e.g. Braude, 2012; Hilton and Slotnick, 2005; Monrouxe et al., 2011; Ng et al., 2015; Pellegrino and Thomasma, 1993), *phronesis* holds the key to understanding the nature of medical professionalism, how it is developed and how ethical decision-making is learned.

Pellegrino and Thomasma (1993) provided what is, perhaps, the most forceful defence of phronesis in medical practice. They hold that rules- or principles-based approaches to medical ethics are ‘too abstract . . . [and] . . . too formularized and far removed from the concrete human particulars of moral choice’ (Pellegrino and Thomasma, 1993: 19). In their conception, *phronesis* plays a crucial part: it is to them ‘medicine’s indispensable virtue’ (Pellegrino and Thomasma, 1993: 84). *Phronesis* provides a crucial link between a doctor’s medical knowledge/reasoning and their moral self, providing the key to resolving a long-standing issue in medicine – how to navigate the competing scientific and humanistic demands of ethical medical practice (Pellegrino and Thomasma, 1993).

Recently, Kaldjian (2014, 2010) has argued that a proper understanding of doctors’ clinical thinking requires seeing good clinical judgement as a form of *phronesis* itself. Developing these themes, Kaldjian reasoned that *phronesis* is needed for the doctor to integrate four different imperatives that may sometimes pull in different directions, these being: scientific knowledge, the patient’s preferences, their own moral view and the priorities of society. Due to these complex factors, developing practical wisdom in doctors takes years of practice combined with learning by example from experienced clinicians.

Despite the great deal of attention that *phronesis* has received in the literature on medical ethics, discussions of phronesis in medicine have tended towards the *theoretical* rather than on the *empirical.* Debate has centred on what the concept itself means (see Kristjánsson, 2015 for an exploration of these debates). Previous work on virtue approaches to medical ethics (including *phronetic* approaches) identified some early-stage research on virtue in medicine generally (Kotzee and Ignotwicz, 2015). However, a further survey of the literature conducted by ourselves identified only 17 qualitative empirical studies on *phronesis* or practical wisdom in medicine in the last decade. While the concept of *phronesis* has had more of an impact in nursing (12 published studies), few of the studies in our review focused on the development of phronesis in doctors (although, for a notable exception, see Little et al., 2011). Furthermore, these do not consider in detail how *phronesis* can be developed, whether it can be taught or what exactly it can contribute to understanding ethical decision-making in current medicine. Instead, study authors often assert the need for phronesis without providing empirical evidence that it exists or how to learn and implement phronesis in practice.

## The narrative development of phronesis

One thing that the studies reviewed had in common is that they all drew on a narrative approach, asking participants to recount narratives, in their own words, of wise judgements in practice. In theoretical accounts of phronesis, narrative also plays an influential role in how phronesis is thought to be developed. Aristotle regularly stressed that ‘ethics’ is not an exact science. Questions regarding what is right and wrong (morally) cannot be answered with the precision that we would associate with a science such as physics or chemistry, because ethics is context-specific. For Aristotle, we can only understand actions and their actors as right or wrong by having a deep understanding of all the relevant facts about the situation, meaning the context in which they acted. (For Aristotle’s own explanation, see, for instance, NE I, 7 1098a, 27-35 and NE II, 2, 1104a, 1–5. For discussion see Irwin, 2000 and Hughes, 2013). Without knowledge of the specifics of the situation in which a person acted, as well as their motivations in acting, it is impossible to know if the actor acted with or without virtue and, therefore, rightly or wrongly.

The most natural way to come to know the specifics of a situation that influenced a decision to act one way or another is to ask the actor to account for themselves which, in effect, requires them to tell a story or offer a narrative about the context and the decision they took. Simply put, the description of virtuous behaviour naturally involves the telling of something like a story. And when one needs to provide a description for another person regarding what is the virtuous thing to do – the kind of description that one needs to supply in *teaching* about virtue, for instance – narrative is a very natural vehicle to use. For this reason, the teaching of virtue is often accomplished through the telling of stories.

Narrative and storytelling are thought to be crucial to the development of phronesis, as they play an integral part in communicating stories about exemplars and their actions. Storytelling holds an important role in moral education as stories ‘engage our motives much more than abstract theories, and narratives are crucial to shaping our vision of a good life’ (Zagzebski, 2013). In addition to informing, storytelling can be understood as a ‘model for human action and meaning’ (Schultz and Flasher, 2011) – that is, stories can give some kind of template for what to do. The social sciences in particular have highlighted the important role that storytelling plays in moral education within a given community. Like the exemplars prized in virtue ethics, stories are not just sets of facts, ‘they are organising devices through which we interpret and constitute the world’ (Lawler, 2008). Storytelling to teach morality is as much a part of the social world as it is virtue ethics.

Let us look more closely at the mechanics of how narratives work to drive moral education in a virtue ethics framework. Narrative approaches to the acquisition of virtue argue that people understand and learn virtues primarily through firsthand, narrative accounts of the actions of moral exemplars, that is, people who exemplify virtue (Zagzebski, 2013). As Zagzebski’s (2017) ‘exemplarist’ account of the acquisition of virtue suggested:

[w]e learn through narratives of fictional and non-fictional persons that some individuals are admirable and worth imitating. (p. 15)

For Zagzebski, we cannot describe what, exactly, makes a person a moral exemplar – moral exemplars do not all have certain features that we can pick out. Instead, moral exemplars are picked out through *ostension* (pointing out). As Zagzebski (2017) wrote:

basic moral terms are anchored in exemplars of moral goodness, direct reference to which are foundational in the theory. Good persons are persons *like that*, just as gold is stuff *like that*. Picking out exemplars fixes the reference of the term ‘good person’ without the need for descriptive concepts. (p. 15)

Zagzebski held that we do not understand what it is to be morally good first and then understand that a particular person is morally good. Rather, we can see that a person is morally good and understand morality on the basis of being like that person. For instance, we do not first understand what honesty is and then see that Lincoln is honest; instead, honesty is being like honest Abe.

Zabzebski also argued that we have a particular feeling for these exemplars: admiration. This feeling of admiration is shaped through the stories that people tell of real and fictional moral exemplars, so admiration is ‘educable’ (Zagzebski, 2017). Because we admire moral exemplars, we try to learn from them and, as a result, ‘emulate’ them. For Zagzebski, this is what moral practice comes down to: imitating exemplars in how one acts.

## Studying phronesis: creating a hybrid social science–humanities-based methodology

Starting from Zagzebski’s exemplarist understanding of the acquisition of virtue, we were interested to study the development of phronesis among junior doctors. Given the importance of narrative in both theoretical and empirical work on phronesis, it became clear that a hybrid methodological approach would be required if we were to capture the empirical data necessary to examine the importance of phronesis in learning to make ethical decisions in medicine, and the role that storytelling may play in the development of that phronesis. To reflect the humanities and social science frameworks within which our research is situated, a qualitative research design that focused on interviews was chosen for this project. This design reflects both the importance of qualitative research to empirical ethics and the emphasis placed on narrative in existing work on phronesis. Qualitative research epistemologies view knowledge as situated and contextual, and interviews allow for a ‘thematic, topic-centred, biographical or narrative approach’ that is in line with interpretivist ontology (Mason, 2002). Interpretivist approaches argue that people can and do tell others about their lives (Brewer, 2000) in the same way that Boje (1991) and Zagzebski (2013, 2017) argued that people share stories about virtuous people and their actions as sense-making activities. One way interpretivist research accesses these lived experiences is through semi-structured interviews, which involve an interactional exchange of dialogue that is relatively informal in style, as well as fluid and flexible, allowing space for the storytelling that serves as both an educational tool and social resource (Mason, 2002).

Boje (1991) argued that this kind of storytelling occurs predominantly in conversations, which involve ‘listeners’ (p. 107). In this project, the interviewers have assumed the role of ‘listeners’ by soliciting stories about virtuous or wise decision-making from the interviewees. As with any qualitative study involving interviews, these listeners are also ‘co-producers with the teller of the story performance’ and thus participate in the storytelling by enabling the telling of the story by the storyteller (Boje, 1991: 107). In this way, our research is not only examining the stories that get told about phronesis and virtuous behaviour, but we are actively participating in the storytelling that we argue is a necessary part of phronetic education in the wider context.

Combining the humanities and the social sciences in our methodology is also part of a growing trend towards studying medical ethics empirically (Paton, 2017, 2018). This growing realisation of the role social sciences play in illuminating practice and informing theory in medical ethics is often referred to as ‘empirical ethics’ or ‘sociological bioethics’ (Haimes, 2002; Paton, 2017). However, as we made clear earlier, few studies have empirically and qualitatively examined phronesis in medicine. We set out to remedy the situation by using methods, methodology and theory from the social sciences, alongside the ethical theory from the humanities to examine and analyse the research questions, making this project part of a new wave in empirical ethics research that goes beyond the use of social science methods only and incorporates valuable social and sociological theory in its design and analysis as well (Paton, 2017).

## Methods

For this study, we conducted semi-structured interviews with second- and final-year medical students, junior doctors (newly qualified doctors) and experienced doctors across three medical schools and hospital governing bodies (called ‘trusts’) in central England over a period of 2 years. Ethical approval was granted at each university and associated trust for the medical schools of Birmingham, Nottingham and Warwick. Participants were recruited through a combination of invitation to participate emails sent through the school or trust and presentations given by the authors during lectures and training. Interviews were audio recorded and transcribed with the written consent of the participants. In this article, we will focus on the interviews with the junior doctors, as this was the longitudinal sample of the research. These participants were interviewed once at the beginning of their first foundation year (their first year after medical school as a doctor) and again during their second foundation year (their second year after medical school as a doctor). In total, 46 interviews were conducted with junior doctors. Twenty-eight participants were interviewed during their foundation year 1, 12 of this initial group agreed to a follow-up interview in their foundation year 2, and 6 were newly recruited participants in their foundation year 2. Transcribed data were analysed by both authors in NVivo using thematic analysis (Attride-Stirling, 2001). Themes were refined through secondary analysis by both authors together, then further analysed with regards to narrative and virtue theory. A number of themes were identified, with storytelling being one of the most prominent and consistent themes across all cohorts and interviews.

## Results

In interviews, participants routinely approached medical ethics narratively. Participants told stories about moral dilemmas they and others had encountered and would often use narrative to illustrate how they understood phronesis, wisdom and wise decision-making. Most often participants used stories to explain ethical dilemmas and, in particular, articulated how hearing and telling stories about ethically difficult scenarios or moral exemplars were their preferred ways to learn how to make ethical decisions and, by extension, develop phronesis. The terms ‘phronesis’, ‘practical wisdom’ and even ‘wisdom’ were not frequently used by interviewees, and many participants had never come across the term ‘phronesis’ before. However, the concept of phronesis and the development of phronesis through experience (both their own and the ‘vicarious’ experience gained from listening to others’ stories) were readily acknowledged by all participants as the primary way doctors learn to make ethical decisions. Interviewees also articulated an understanding of wise/unwise doctors and wise/unwise decisions that they had witnessed or heard about.

Three major themes emerged from the analysis that captured the importance that storytelling has to how doctors use storytelling as part of their ethical education and phronetic development:

1. Learning medical ethics through storytelling.
2. Developing phronesis through storytelling and ‘story-listening’.
3. Passing on/teaching phronesis through ‘phronesis narrative’.

We would like to turn to each of these themes now to explain the role of storytelling to the development of phronesis, such that it facilitates ethical decision-making in medical practice.

### Learning medical ethics through storytelling

Participants rarely spoke about the procedure or the ethical guidelines that they had learned during their training. Instead, when asked to discuss ethics, narrative was the preferred way that participants communicated important ethical concepts or lessons they had learned that they now used in their own practice.^1^

Just from casual conversations with a lot of the medical officers you can tell that these ethical issues are actually really memorable and they will talk about it [. . .] and they’ll say oh we had this situation and it was really difficult to work [out] what’s the best to do and yeah you can see that that’s going to be really prominent in our practice. (A109)

Participants were particularly keen to discuss moral dilemmas they and their colleagues had encountered, and the lessons they had taken from them.

[. . .] so ethical issues that people haven’t come across before and you think, ‘Oh, I’ve never even considered that as an issue’. It’s good to hear from other people to think, ‘Right, well, how did you deal with that?’ (C107)

A vital element of this was the sharing and discussing of the stories between colleagues, and many participants highlighted how an important aspect of learning through stories was the collegial element of doing so with a fellow healthcare professional.

We have a chat about things [. . .] saying, ‘Well, I’m not quite sure how to approach this’, then you may have, ‘Oh, well, this is how I did it in the past’, or, ‘This is how I’ve seen somebody else do it really’. . . . That taught me quite a lot. (B105)

But I think those are the ones where I will be speaking to [. . .] either a colleague or a nurse [. . .] talk about what they would do in this situation [. . .] I am still quite open to taking on other people’s opinions in my own decision making, because I feel like there is a lot of room for more knowledge and things in where I am right now [. . .]. (A101)

### Developing phronesis through storytelling and listening to stories

While participants rarely used the term ‘phronesis’ in interviews, they often described the concept and its development when discussing how storytelling and listening to stories helped participants develop a bank of experience. This was a recurrent theme articulated by the participants in their initial and follow-up interviews. This experience did not necessarily have to be developed through personal experience, but through the hearing of stories of other people’s experience. Together, these stories formed a quasi-collective library of experience for junior doctors from which they often drew to seek advice, make ethical decisions and take action.

. . . because part of wisdom does have to be listening to other people . . . (A101)

I don’t think anyone is born with the ability to make wise decisions. I think it’s, a lot, based on experiences, and through observations of other cases; and review and reflection of other cases. (A111, follow-up)

[. . .] I don’t think intelligence and wisdom are the same thing; you can be intelligent, smart, but having wisdom means taking your time, taking a step back, having a broader view of the problem, thinking beyond your immediate self. (C104, follow-up)

Talk to your seniors about why they made that particular decision that time. [. . .] It’s something that I don’t think can be taught in just a lecture, that over time you can pick up some of the reasoning that other people have been making. (C107, follow-up)

Wise and unwise decisions were frequently discussed and participants often told stories about wise or unwise decisions they had witnessed, as well as wise and unwise doctors they had worked with.

I think storytelling, whether it’s a case that happens around the table that you look at a patient, or something that’s been more informal in life probably forms part of your memory. Essentially it forms a bank of wise and unwise decisions. (B104)

[. . .] I feel like my GP trainer makes a lot more decisions based on his practical wisdom from similar cases and things rather than necessarily doing lots of tests which sometimes feel fairly pointless in a hospital setting. (A108, follow-up)

Participants also regularly stressed that learning to be wise was a matter of experience that builds up over time.

I feel as though med school taught me quite well, ethics, four principles, all that kind of stuff – sort of more formal [. . .] It’s more suited to a classroom [. . .] I think the phronesis [. . .] I think that pertains a lot more to being a doctor; and I think being able to make those on-the-hoof decisions [. . .] the practical wisdom, where you’ve had years of ethical training behind you, that is then linked up to your actual practical experience, and your expertise in that area. I think phronesis more aptly describes what a doctor does on a day-to-day basis. (Al05, follow-up)

In particular, participants shared stories of how moral exemplars, who always had more experience, dealt with difficult ethical dilemmas, which in turn helped them to act wisely in the future. These stories were sometimes first-hand accounts of something the participants had witnessed, but were often retellings of the story after it had been shared by the actor themselves, or through other colleagues.

. . . in my last job of F2, my medical consultant, one of the respiratory physicians, he was a really, really solid role model. He’d make a decision; you could see why he’s made it, or even if you couldn’t see why he’s made it, it makes sense when he explains it to you, and yeah, those are people I really look up to, medically. (A105, follow-up)

I think we see our seniors, often, as role models; and I think people are often very quick to make decisions about their senior consultant, as to whether they’re a good role model to have, or whether they’re not; and people do role-model. I think most junior doctors, from my experience, do role-model themselves off their consultants who they think are particularly good [. . .] ‘That consultant’s really good at that. I want to be like that when I grow up, if you like’. (B107, follow-up)

There was one doctor I was with, actually – this is in medical school – who really struck me as wise. [. . .] I sat in with him for one clinic, and at the end of it, he was explaining [. . .] how with each patient who comes in the door, he’s trying to gauge what kind of doctor would be most helpful for them, and then to fill that role for them. And so, like, some people, he would say, are looking for more of a kind informal, sort of an equal kind of a chat; and you get some people looking for more of, you know, just someone who will tell you the answer, and say, ‘This is what we need to do’; or one might want you to joke around, and one might want to be very serious –and he’s kind of feeling for that, always, and trying to feel that. I just thought that was really, really lovely. (B108)

Some interviewees did consider whether, next to stories about exemplars, there are some other ways to develop phronesis. The most obvious alternative would be to try and codify what a wise decision is in some kind of procedure. It is notable that when exploring this possibility a number of interviewees quickly pointed out the shortcomings of having some kind of codified procedure to encode how to make a wise decision. Again, exemplars with experience were appealed to by the participants as a way to adjudicate difficult ethical dilemmas.

I like guidelines, I think they’re really useful especially if you’re the junior and I think in a way you’ve got to stick to the guidelines but there’s difficult situations where guidelines don’t help you because it’s either such a specific situation that you can’t go by the guidelines and you’ve got to speak to somebody with experience. (A110, follow-up)

Many participants explained that more generally guidelines themselves often do not help in making a clear decision, as the guidelines themselves need to be interpreted, and this is a matter of judgement.

I suppose things like at what point to stop treatment for someone who’s got an infection with very advanced malignancy. You can’t have a tick box – ‘If they’ve got this, this, or this, you should stop’. (B107, follow-up)

When faced with this problem, interviewees regularly said that it is a matter of experience how to interpret and implement a guideline.

### Passing on/teaching phronesis through ‘phronesis narrative’

One of the most interesting ways that participants used storytelling and narrative to develop phronesis was through the use of what we call ‘phronesis narrative’. There were stories that participants told us about how they learned to use better judgement and be more wise. Often, these narratives were told as a form of self-confirmation or self-learning of lessons learned from the participant’s own experience in developing phronesis. However, they also acted as part of the life cycle of phronetic development: in the act of retelling these stories participants were contributing to their own phronetic development, and passing on stories for the development of others by imparting their experience and growing wisdom on a subject.

[. . .] we often have to assess capacity, do [patients] understand the risks [. . .] which is often me doing it.[ . . .], as a fairly new doctor, I think our ethical training has been much better than the older doctors. And, I think we are much more knowledgeable about the process of assessing capacity, and understand, ethically, what’s expected of us. So, that happens really regularly, and it’s often the Juniors who are stepping up to do that capacity assessment, rather than the Registrars and the Consultants. (A109, follow-up)

So in one situation in [the emergency department] there was a girl that had come in saying that she had been assaulted and I did the history [. . .] and there was someone else in the room, a partner and then when I discussed it the nurses had some concerns and said we think it’s a safeguarding issue because they recognise her and says she comes in repeatedly with lots of different injuries and things. And I went and spoke to one the consultants about it and I hadn’t actually taken the girl away from the partner or asked the partner to leave the room to speak to her in private, I’d just spoken to her while he was in the room. And immediately when the consultant came he asked the partner to leave while he had a private chat and then just asked her directly if it was domestic violence and that was something I learned quite quickly. Like to separate, if you are thinking of domestic violence or something like that just separate the patient away, which is something, it sounds obvious but at the time I just hadn’t thought about it [. . .]. (C102)

## Discussion

Storytelling of difficult situations and of moral exemplars took a wide variety of formats in the data, from informal hallway chats, to professional development meetings, to formalised rounds. In medicine, good or virtuous action is defined as the kind of action a good or virtuous doctor would take, and ‘after the fact’ accounts, like those described earlier, are common practice. Our data confirm that doctors not only share stories of events with one another (Montgomery Hunter, 1991) and learn and develop professionally from this storytelling, but that the type of story shared has a significant influence as well. In particular, when stories are told about ethical dilemmas in medical practice, the learning takes on a moral dimension and these stories become *phronesis* narratives that facilitate medical ethics education and development.

The important role of storytelling in moral learning has been previously recognised in various fields that reflect the social science–humanities hybrid roots of this study, but have never been explicitly brought together to show the link between storytelling about ethical dilemmas and learning how to act ethically as a doctor. Philosophical work on narrative recognises that narrative is one crucial way of understanding human action and meaning (Schultz and Flasher, 2011). Understanding the ethical dimensions of human action, in particular the normative judgements placed on human action, is often achieved through narrative. While narrative as a ‘constitutive aspect of ethics’ is often denied in favour of an objectivist view of ethical decisions and medical responsibility in medicine, this denial does not negate the importance that narrative has in understanding medical ethics (Schultz and Flasher, 2011: 396).

However, ignoring the important role of narrative in providing moral explanation only serves to ‘conceal the “lived” dimension of meaning in ways that alienate, marginalise, and estrange patients and providers from themselves and their worlds’ (Schultz and Flasher, 2011: 396). As a result, instead of using narrative to interrogate ethical decisions and examine the ethical dimensions of medicine, it is often viewed as a value-neutral tool that can do little more than ‘provide a record of the facts of the illness’ (Schultz and Flasher, 2011: 396). Ignoring narrative in ethics, and in particular in medical ethics, is an extension of the applied bioethics legacy, which attempts to view medical ethics as aforementioned interpretation and universally applicable, while disregarding the ever changing complexities of the medical realm and the individual context within which each ethical decision is made (Paton, 2017).

Instead, as Schultz and Flasher (2011) argued, it is necessary to recognise that ‘appeals which guide the exercise of “right reason” in medical and ethical decisional activity are, themselves, already shaped by narrative discourse’ (p. 401). Whether medical science and objectivist accounts of medical ethics want to acknowledge the role of narrative in medicine is irrelevant, as narrative is a fundamental part of the medical institution. As Kathryn Montgomery Hunter (1991) argued in Doctors’ Stories, medical knowledge is inextricably rooted in narrative; without narrative, in particular patient narrative, it would be impossible to take patient histories and share experiences of previous cases, both of which are necessary features of treating patients. Medical knowledge needs storytelling as it is a ‘special way of knowing’, and medical knowledge without narrative is incomplete (Schultz and Flasher, 2011: 399). Our data echoed these same arguments, with participants continually highlighting the important role that narrative had in their moral learning. Participants provided examples of how they experienced narrative as a fundamental part of practicing not just medicine, but ‘good’ medicine that they believed was also ethically sound. Narrative was considered by participants to be a crucial element of it as ‘the way [. . .] providers and patients understand the realities of health and illness is determined by how the story is articulated’ (Schultz and Flasher, 2011: 404).

### Moral exemplars and phronetic development

An important articulation of the story is the moral exemplar; however, previous work has ignored the role of the exemplar in phronetic development in favour of more generalised discussion of phronesis in medicine. In the data, moral exemplars and the stories about them were a prominent source of phronetic development and moral learning. Participants consistently told stories about moral exemplars and explicitly discussed how they used moral exemplars as compasses for their own actions.

Further discussion of Zagzebski’s ‘exemplarist theory’ of the nature of virtue, which gives a central role to exemplars, helps us to understand the importance exemplars had to participants. As we have previously argued, the motivating emotion that drives human action is admiration (Zagzebski, 2013). Like Aristotle’s virtuous man, an exemplar is a ‘paradigmatically good person’ who inspires admiration and as a result those observing the exemplar wish to imitate them (Zagzebski, 2013: 201). By admiring the virtuous traits of exemplars, it is possible to develop a similar character to them, thus becoming virtuous ourselves. Zagzebski (2017) wrote:

The model I am proposing starts with admiration of an exemplar, which leads to an imaginative ideal of oneself, which in turn produces emulation of the exemplar’s motives and acts. The moral learner does the virtuous act from a virtuous motive because the learner is emulating someone who does that act from that motive. With practice, the agent becomes disposed to doing acts of that kind from motives of that kind.

As we found in our research, Zagzebski suggested that there is a link between storytelling and moral education through the narrative vehicle of exemplars. As she put it ‘narratives are the primary vehicle for the moral education of the young, the primary way humans of any age develop and alter their moral sensibilities’ (Zagzebski, 2013: 196). This holds true for those training to become medical doctors. Stories are also helpful in precipitating action as they ‘capture the imagination, and elicit emotions that motivate action’ (Zagzebski, 2013: 196). We also found it easy to identify exemplars possibly because, as Zagzebski (2013) pointed out, the practice of identifying them is embedded in our current moral practice as ‘we learn through narratives of fictional and non-fictional persons that some people are admirable and worth imitating’ (p. 199). Storytelling is an essential part of identifying exemplars; they are essentially a narrated observation about the virtues and traits of an individual person, which ‘reveal the necessary features of value by uncovering the deep properties of a good person’ (Zagzebski, 2013: 200). In addition, the use of exemplars is flexible, as exemplars for a given scenario can change given the context, and like phronesis, an individual can be ‘good’ or ‘virtuous’ in different ways, depending on the situation they are observed in (Zagzebski, 2013).

### Phronesis narratives

Participants used stories about exemplars, and stories about ethical dilemmas more generally, in a unique way to reflect on their own actions and reinforce their moral learning through the telling of phronesis narratives: narratives that explicitly laid out how they learned to become wise. Phronesis narratives are an important social phenomenon in moral learning as the social world is ‘storied’ (Lawler, 2008), and phronesis reflects this as it is developed through the sharing of stories. Stories are in all aspects of encounters and are part of how people make sense of their lives and others. Importantly, stories are a contextual source that people use when ‘sense-making’ (Lawler, 2008). This is because life stories, the stories we tell each other about each other, are normative. They contain within them rules that ‘adhere to local (in time and space) intelligibility norms’ from within our own cultures (Lawler, 2008: 33; see also Gergen and Gergen, 1986). Participants used phronesis narratives as part of their sense-making process. By telling phronesis narratives participants were ‘checking’ their actions, while also passing on moral knowledge to others. Just as exemplars are admired for their virtuous traits, these life stories elicit responses based on the existing norms about right and wrong action, through which we can judge others after hearing the stories and further our moral education, making these stories important ‘cultural and social resources’ (Lawler, 2008; Zagzebski, 2013).

One aspect of these phronesis narratives that we argue is a crucial part of understanding the importance storytelling has to moral, phronetic development is that of ‘emplotment’. Emplotment examines not just the detail of the story itself, but the process of storytelling as well. As Lawler (2008) so carefully argued, emplotment considers ‘the narrative in its social context: stories completed [. . .] in the circulation of relations between story, the producer of the story, and the audience for the story, in the context of local rules for what constitutes a meaningful story’ (p. 33). In short, emplotment examines what the story ‘does’, by not only being told, but simply existing as a story to be told or that has been told. We argue that one of the things that storytelling ‘does’ is promote moral education through the storytelling of exemplars, their character and actions. Stories are told within the context of ‘local rules’, and as such they can carry normative value about right and wrong actions, and the people who carry them out, within a particular culture. This argument is further supported by the articulation of interviewees’ that ethics policy and guidelines were better taught (and understood) through narratives about difficult ethical situations, than through the inflexible tick-box structure they were formally taught in training.

This is not to say that the context of the stories, the character, action and plot, is not also an important part of storytelling. Plot in particular helps the story ‘do’ what it ‘does’ by showing the point of the story, which in the case of exemplars, moral education and phronesis is about revealing the virtuous character of an exemplar. Lawler (2008) argued, and we agree, that narrative must have a point, as plot is ‘a central feature of narrative [. . .] it brings together different events and episodes into a meaningful whole’ (p. 35). Plot not only assigns meaning to stories but also an essential way that people engage in the learning of morality through storytelling as both the narrator and the audience participate in linking the plot together ‘through a shared cultural understanding that *these* events have a place in *this* narrative’ (Lawler, 2008: 36). The story of the exemplar serves a particular function when told in a particular context within the social/cultural environment in which the story is shared. As such, part of creating and using the narrative as a resource is to listen to it and consider its significance in action. In this way, stories can over time almost become exemplars in and of themselves if they carry significant narrative about how to act.

The development of stories and narratives into entities in and of themselves is not unprecedented. The sharing of patient illness narratives has been viewed in a similar light, with Frank (1995) arguing that as these narratives are shared they develop a power of their own through their retelling, becoming an ever-widening ‘circle of shared experience’ (p. xii). Both Frank and Kleinman classically argued that this kind of storytelling is an ethical act in itself: arguing that by telling the story the narrator assumes normative responsibility for that story (Frank, 1995), as the dialectic between teller and listener is an ‘opportunity for moral education’ (Kleinman, 1988: xiv). Similarly, phronesis narratives are not just the sharing of experience, but carry with them a normative impetus to pass on these stories of a way of growing the bank of experience, thus furthering the development of phronesis and the continuing of a medical moral education.

## Conclusion

Given storytelling’s importance as a social resource, which dates all the way back to Aristotle, it is unsurprising that ‘narratives always and necessarily build in attempts at understanding’ (Lawler, 2008: 36). The telling of and listening to stories can be considered deliberate, normative actions, as there are ‘consequences of storytelling – for those who tell the stories and for those who study them’ (Czarniawska, 2004: 37; Frank, 1995). Stories guide actions (Somers and Gibson, 1994), and when stories guide action, the act of storytelling is normative in that we are engaging in an activity that has important consequences within a community. As Reissman argued, ‘storytellers interpret the world and the experience in it: they sometimes create moral tales – how the world should be’ (Riessman, 2005: 1). This is particularly true of the role that storytelling has in influencing decision-making. Boje (1991), like Lawler and others, argued that stories are the ‘preferred sense-making currency’ of human relationships and are used to help decision-making by helping listeners to avoid ‘repeating historically bad choices’ while inviting listeners to consider the value of ‘the repetition of past successes’ (p. 106). Storytelling is a powerful tool with which to promote moral education and the development of phronesis and thus deserves a more prominent role in the medical ethics education and training curriculum.

## Notes

1. Interviewees are identified here using a letter to denote the hospital or medical school site (A, B or C), the number 1 to denote the interviewee is in their foundation year 1, and subsequent numbers have been randomly assigned to distinguish between speakers. Where ‘follow-up’ is noted, this indicates that the participant was interviewed twice, the second time during their foundation year 2.
